# THE TRAJECTORIES OF COGNITION AND FUNCTIONAL LIMITATIONS OF CENTENARIAN SURVIVORS

**DOI:** 10.1093/geroni/igac059.1206

**Published:** 2022-12-20

**Authors:** Gina Lee

**Affiliations:** Iowa State University, Ames, Iowa, United States

## Abstract

The purpose of this study is to explore cohort differences in the trajectories of cognition and functional limitations among oldest-old adults. Using 1992-2018 data from the Health and Retirement Study, participants included those who survived to at least 98 years (N=494). The study included three cohorts (i.e., 1890-1900, 1901-1910, and 1911-1920) predicting intercept and slope of cognition and functional limitations. Two separate latent growth curve models were computed, and models of cognition, χ2(5)=6.935, p=.226 and functional limitation, χ2(7)=7.743, p=.35 fit the data well. There was a significant decline in cognition levels and a significant increase in functional limitation over time. When adding cohort, results indicated that older cohorts were more likely to have a decrease in cognition and increase in functional limitations compared with the younger cohort. Future research should investigate trajectories of other health variables for a better understanding of health changes of centenarians.

